# Expert Consensus Guidance on the Management of Chemotherapy-Induced Nausea and Vomiting: An Indian Perspective

**DOI:** 10.7759/cureus.84070

**Published:** 2025-05-13

**Authors:** Boman Dhabhar, Prabrajya N Mahapatra, Vamshi M Krishna, Jatin Sarin, Tara Chand Gupta, Aseem Samar, Bivas Biswas, Aditya Murli, Rajesh Kota, Suyash Bharat, Richa Tripathi

**Affiliations:** 1 Department of Oncology, Fortis Hospital Mulund, Mumbai, IND; 2 Department of Oncology, Apollo Cancer Centre, Kolkata, IND; 3 Department of Oncology, Asian Institute of Gastroenterology, Hyderabad, IND; 4 Department of Oncology, Chandigarh Cancer and Diagnostic Centre, Chandigarh, IND; 5 Department of Oncology, Bhagwan Mahaveer Cancer Hospital and Research Centre (BMCHRC), Jaipur, IND; 6 Department of Medical Oncology, Apollo Multispecialty Hospital, Kolkata, IND; 7 Department of Oncology and Hematology, Apollo Cancer Centre, Bangalore, IND; 8 Department of Oncology, American Oncology Institute, Vijayawada, IND; 9 Department of Medical Affairs, Zydus Lifesciences Ltd, Ahmedabad, IND

**Keywords:** antiemetic prophylaxis, cancer care, chemotherapy-induced nausea and vomiting (cinv), clinical guidelines, indian consensus, modified delphi method

## Abstract

Chemotherapy-induced nausea and vomiting (CINV) significantly impact patients' quality of life and treatment adherence, with high incidence rates despite the use of antiemetic prophylaxis. While international guidelines provide recommendations for managing CINV, the diverse healthcare landscape in India necessitates tailored, region-specific guidelines. This study was conducted to develop and validate consensus-based clinical statements on CINV management tailored to the Indian context, aiming to enhance the quality of cancer care across the nation by aligning international insights with local experiences. A comprehensive literature review and a consensus-based methodology were employed. 150 Indian oncologists participated in developing 14 clinical statements through the modified Delphi method. Two rounds of voting were conducted to assess agreement levels, categorizing the statements into the following three categories: consensus, near or fair consensus, and no consensus. Consensus was achieved for 11, while three received near or fair consensus. In areas where data or agreement was lacking, expert opinions were incorporated to supplement the findings. One of the findings from this study indicated strong consensus for the use of netupitant and palonosetron along with dexamethasone as the preferred CINV prophylactic regimens in an Indian setting. The consensus statements developed in this study will assist Indian oncologists in making informed, tailored decisions regarding CINV prevention and management across diverse healthcare settings.

## Introduction and background

Chemotherapy-induced nausea and vomiting (CINV) represent a significant challenge in cancer therapy, impacting patients' quality of life and treatment outcomes [1]. Additionally, patients with CINV are discouraged from completing their treatment regimen, leading to reduced adherence to the therapy and, thus, the outcomes [2]. The incidence of acute CINV during the first 24 hours of starting moderately emetogenic chemotherapy (MEC) or highly emetogenic chemotherapy (HEC) varied between 30% and 90% in the absence of antiemetic prophylaxis [3,4], while that of delayed CINV was found to be 28-50% among patients taking MEC or HEC [5]. This data underscores the need for effective antiemetic methods. Furthermore, despite adherence to guideline-recommended antiemetic regimens, breakthrough CINV impacts nearly 13-24% of patients, highlighting the ongoing deficiencies in management and the need to enhance prophylactic strategies [4].

Over the past few years, international guidelines from esteemed organizations such as the Multinational Association of Supportive Care in Cancer (MASCC), the European Society for Medical Oncology (ESMO), the American Society of Clinical Oncology (ASCO), and the National Comprehensive Cancer Network (NCCN) have provided valuable recommendations for CINV management [6-8]. Agents such as serotonin receptor antagonists (5HT3-RA), dopamine (D2) receptor antagonists, neurokinin 1 receptor antagonists (NK1-RA), and corticosteroids are recommended to help prevent CINV [6]. However, these international guidelines are not tailored for the Indian population, which has varying patient demographics and healthcare resources [9]. They have been adapted as is for clinical practice in India, and there are no specific guidelines or recommendations from India. The applicability of these guidelines in the Indian context may be influenced by factors such as cultural preferences, resource constraints, and variations in clinical practice [9,10]. As a result, developing region-specific guidelines for CINV will be effective, as they may consider patient risk factors, affordability, sociocultural influences, and clinical oncology practices in the nation.

This position paper, therefore, attempts to synthesize consensus statements and research findings from the shared experiences of Indian oncologists on CINV management. By consolidating insights from international guidelines, research articles, and the experiences of Indian oncologists, this document aimed to provide a nuanced understanding of CINV management in India. The quality of treatment for cancer patients throughout the country might be raised by establishing uniform protocols that consider the reality of clinical practice via professional consensus-building. We aimed to bridge gaps in understanding and highlight widespread practices in managing CINV across the country.

## Review

Methods

A detailed systematic literature review was conducted, including randomized clinical trials, systematic reviews, and meta-analyses in databases such as MEDLINE (via PubMed) and Cochrane-indexed databases, as well as international guidelines, especially NCCN and ASCO guidelines [6,11]. Based on the literature review, 14 consensus-based clinical statements were formulated that focused on crucial aspects of CINV management. Of the 14 statements, 13 were based on research articles and guidelines, while one clinical statement was developed as a hypothesis based on the practical experiences of the clinical oncologists. These statements were then validated against the literature, and initial responses were gathered from the core expert team, which consisted of nine oncologists and the medical affairs team. The initial inputs were achieved via the first round of voting from 95 Indian oncologists from various parts of India via one-on-one interaction using a virtual medium between January 2022 and September 2023.

The Modified Delphi methodology was applied to achieve consensus on the votes achieved via the first round of voting [12]. Following the first round of voting and its analysis, a second round of voting was conducted among 55 Indian oncologists through a virtual medium to assess the cumulative consensus on the 14 clinical statements. The second round of voting was conducted among 55 Indian oncologists from September to December 2023. The response of the oncologists to the 14 clinical statements was recorded on a five-point Likert scale. The scale consists of the following points: strongly disagree (score 1), disagree (score 2), neutral (score 3), agree (score 4), and strongly agree (score 5). The consensus-based statements were classified into distinct categories based on the level of agreement among the experts (Table 1).

**Table 1:** Responses received from the Indian oncologists for the clinical consensus statements. *Consensus: statements receiving a mean and median score of ≥4 were regarded as having achieved a consensus agreement. **Near or fair consensus: statements with a mean and median score of 3 to <4 were deemed to have reached a near consensus agreement. These statements may be influenced by institutional and regional clinical practices. ***No consensus: statements failing to meet the criteria for either consensus or near consensus were categorized as having no consensus and are marked with three asterisks. HEC: highly emetogenic chemotherapy; MEC: moderately emetogenic chemotherapy; LEC: low emetogenic chemotherapy; CINV: chemotherapy-induced nausea and vomiting; NK1-RA: neurokinin-1 receptor antagonist; 5HT3-RA: 5-hydroxytryptamine type 3 receptor antagonist; NEPA: fixed-dose combination of netupitant and palonosetron; NCCN: National Comprehensive Cancer Network; ASCO: American Society of Clinical Oncology

No.	Expert consensus recommendation	Mean score	Median score	Level of evidence and grade of recommendation
1.	“Classification of intravenous chemotherapeutic agents by NCCN guideline 2022 into HEC/MEC/LEC/minimal is comprehensive”	4.20*	4.0*	1A
2.	“One of the common reasons for antiemetic treatment failure in CINV management was that the emetogenic potential of chemotherapy was underestimated, and undertreated”	4.11*	4.0*	2C
3.	“Patients who get CINV prophylaxis with NK1-RA + 5HT3-RA + dexamethasone ± olanzapine during 1st cycle of HEC/MEC chemotherapy, in subsequent cycles, anticipatory CINV incidence is less”	4.33*	4.0*	1C
4.	“Benzodiazepines, a type of anxiolytic medication, are to be used for anticipatory nausea and vomiting”	3.75**	4.0*	1A
5.	“Patients who receive HEC - for controlling acute CINV (day 1) should be treated with triple combination therapy containing 5HT3-RA, dexamethasone, and NK1-RA”	4.42*	5.0*	1A
6.	“Patients who receive HEC for controlling acute CINV (day 1) should be treated with four-drug combination therapy containing 5HT3-RA, dexamethasone, NK1-RA, and olanzapine”	4.03*	4.0*	1A
7.	“Patients who receive HEC - palonosetron is the preferred 5HT3 antagonist in triple drug combination”	4.24*	4.0*	1C
8.	“Patients who receive HEC - netupitant is the preferred NK1 antagonist in triple-drug combination”	4.25*	4.0*	1C
9.	“Patients who receive MEC - for controlling delayed CINV, triple therapy with NK1-RA improves outcome”	4.13*	4.0*	1A
10.	“Netupitant + palanosetron is more effective than the 3-day aprepitant-based regimen in “preventing significant nausea”, which is the most difficult-to-control symptom with cisplatin-based HEC, in the overall phase (0 to 120 hours) following chemotherapy”	4.31*	4.0*	1B
11.	“Netupitant-palonosetron, efficacious with standard dosing of 1 dose on day one of a three-day HEC regimen”	4.35*	4.0*	1B
12.	“For patients who are intolerant to corticosteroids, CINV prophylaxis following MEC or HEC administration, a single dose of NEPA plus dexamethasone sufficiently controls acute and delayed nausea/vomiting.”	4.12*	4.0*	1B
13.	“If patients cannot tolerate dexamethasone, consider replacing it with olanzapine.”	3.61**	4.0*	2A
14.	“With 90% striatum NK1 receptor occupancy with netupitant lasting for 96 hours, netupitant dose should be repeated on day fourth or fifth day of a 5-day HEC regimen (hypothesis)”	3.14**	3.0***	2C

Statements receiving a mean and median score of ≥4 were regarded as having achieved a consensus agreement. Statements with a mean and median score between 3 and <4 were deemed to have reached a near consensus agreement, acknowledging that institutional and regional clinical practices might influence such statements. Statements that did not meet the criteria for either consensus or near consensus were categorized as having no consensus.

After collecting the consensus from oncologists, the responses were aggregated, and descriptive statistics were applied to derive a representative opinion for each question. The mean and median scores of the responses to the 14 clinical statements were calculated. In cases where data or consensus seemed insufficient or fragmented, expert opinions were asked to provide a more rounded perspective and to fill in the potential gaps in the consensus. Guyatt et al.'s two-level grading system served as the basis for the level of evidence and recommendation strength (Table 2) [13]. Besides these 14 statements, the experts were also asked for their choice of drug for anticipatory nausea and vomiting.

**Table 2:** Grading system on the basis of level of evidence and recommendation strength. Grading system adapted from Vaid et al. [9]. This is covered under the terms of the Creative Commons Attribution License (CCBY), which permits reuse, distribution, and reproduction in other forums, provided the original authors and source are credited.

Grade	Quality of evidence and recommendation strength
1A	High-quality evidence - strong recommendation can apply to most patients in most circumstances without reservation
1B	Moderate-quality evidence - strong recommendation can apply to most patients in most circumstances without reservation
1C	Low- or very-low-quality evidence - strong recommendation but may change when a higher-quality evidence becomes available
2A	High-quality evidence - weak recommendation and best action may differ, depending on circumstances, patients, or societal values
2B	Moderate-quality evidence - weak recommendation and best action may differ, depending on circumstances, patients, or societal values
2C	Low- or very-low-quality evidence - very weak recommendation, other alternatives may be equally reasonable

Results

**Figure 1:**
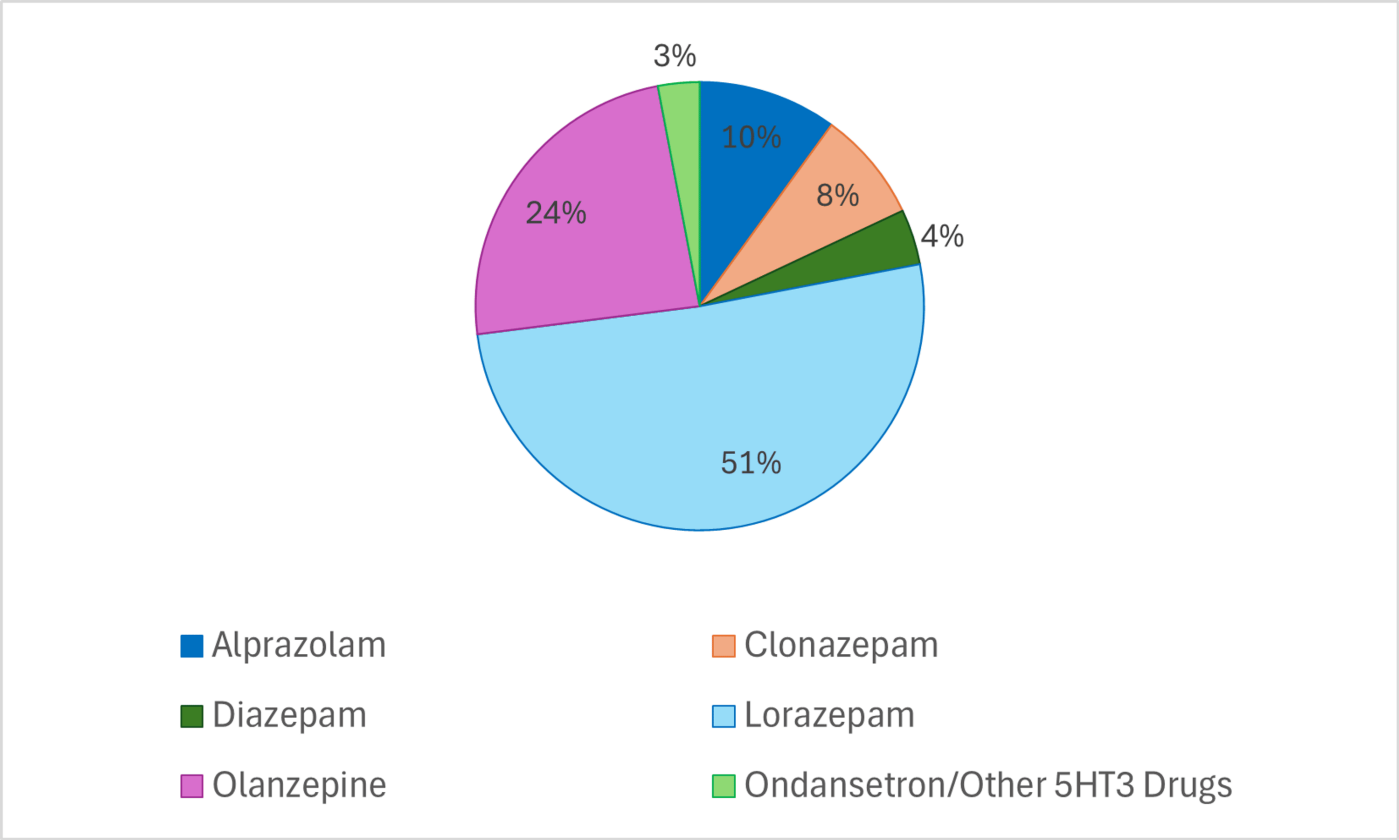
Preferred agent used for anticipatory nausea and vomiting. CINV: chemotherapy-induced nausea and vomiting; HEC: highly emetogenic chemotherapy; MEC: moderately emetogenic chemotherapy; NK1-RA: neurokinin-1 receptor antagonist; 5HT3-RA: 5-hydroxytryptamine receptor antagonists

The authors and experts involved in the consensus process conducted a comprehensive review of the available literature. Additionally, recommendations from international guidelines such as NCCN and ASCO on the management of CINV were also consulted. The mean and median scores of the 14 clinical statements are presented in Table 1. Consensus was achieved for 11 statements, while three statements received near or fair consensus from oncologists nationwide (Table 1). When the oncologists were asked for their preferred agent for anticipatory nausea and vomiting, only 72 oncologists responded (Figure 1). The most preferred choice of agent was found to be lorazepam (51%).

Discussion

Currently, Indian oncologists follow international guidelines such as ASCO, ESMO, and NCCN guidelines to manage CINV among cancer patients. However, these guidelines were not prepared keeping the Indian population in context. Variations in biochemical receptors implicated in the brain regulation of CINV, including 5HT3, NK1, and dopamine receptors, across various populations such as Asians, Americans, or Europeans affect both - the manifestation of CINV and the efficacy of antiemetic treatments [14-18]. Considering these biological variances, substantial geographical disparities, and variability in the management of CINV among healthcare providers, there is a need to standardize clinical practice for CINV management across India [19]. Straightforward implementation of international guidelines may be inadequate. This emphasizes the need for India-specific CINV care strategies customized to the distinct genetic and clinical circumstances of the Indian population. Thus, we have conducted an expert consensus based on the experiences of Indian oncologists to bridge knowledge gaps, highlight common approaches in managing CINV, and represent the professional community's overall perspective.

Parenteral Chemotherapy and Its Emetogenic Potential

National Comprehensive Cancer Network 2024 guideline classifies parenteral and oral chemotherapeutic agents into high, moderate, low, and minimal emetogenic chemotherapy [7]. Experts frequently use NCCN guidelines and find the antiemetic chemotherapy classification comprehensive to tailor the antiemetic prophylaxis of their patients. Despite the National Comprehensive Cancer Network (NCCN) and American Society of Clinical Oncology (ASCO) updating their definition of highly emetogenic chemotherapy (HEC) in 2017 to include carboplatin with an area under the curve (AUC) ≥4, some experts still consider it to be moderately emetogenic chemotherapy (MEC).

Antiemetic Treatment Failure in CINV Management

Ensuring prompt control of CINV is crucial for effectively managing symptoms throughout the chemotherapy regimen. Despite advancements in antiemetic therapy, many patients undergoing chemotherapy outside of controlled clinical trials continue to experience nausea and vomiting, suggesting the potential underutilization of evidence-based antiemetic therapy guidelines in real-world clinical practice. Although guideline-adherent antiemetic therapy has demonstrated efficacy in improving CINV control among cancer patients, there seem to be obstacles hindering oncologists from consistently implementing these guidelines. One of the major reasons for such antiemetic treatment failure was reported to be the underestimation of the emetogenic potential of the chemotherapeutic agents [20]. In our study, expert oncologists also reached a good consensus, suggesting that healthcare professionals often “underestimate the emetogenic potential of chemotherapy and hence undertreat.”

Potential reasons for antiemetic treatment failure, as highlighted in the survey done in 300 oncologists, include underestimating the emetogenic potential of chemotherapy and utilizing weaker antiemetic regimens than required. The study revealed that a significant proportion of patients experienced emesis due to actual chemotherapy emetogenicity being higher than expected, with errors in antiemetic administration also contributing to treatment failure. Notably, the use of "weaker" antiemetic regimens, such as monotherapy instead of combinations, was identified as a common reason for emesis in patients. Psychological factors like anxiety and individual sensitivity were also cited as contributing to treatment failure. Additionally, patient non-adherence to prescribed antiemetic regimens, including administration mistakes or missed doses, as discussed by our expert panel that their patients on aprepitant have to take one tablet every day for three days which leads to non-adherence and this emerges as a notable issue impacting the effectiveness of antiemetic treatments [21]. The oncologists in our study also agreed upon the non-adherence of three-day aprepitant regimen and found it to be most difficult to control symptoms with chemotherapy.

Anticipatory CINV

Anticipatory chemotherapy-induced nausea and vomiting (CINV) refers to the occurrence of nausea and/or vomiting before chemotherapy administration, typically triggered by prior negative experiences with chemotherapy [22]. Anticipatory CINV is best managed by preventing CINV during the first course of chemotherapy. Anticipatory CINV may be caused by inadequate CINV control in prior cycles of HEC/MEC chemotherapy. Such uncontrolled CINV may result in poor treatment compliance or even the cessation of chemotherapy, which may be helpful [23]. Thus, achieving optimal control of acute and delayed CINV during the first cycle of chemotherapy with comprehensive prophylaxis may help mitigate the development of anticipatory CINV in subsequent cycles [20,23]. Experts had a consensus, suggesting that managing CINV prophylaxis during the first cycle of HEC/MEC, as per guidelines, i.e., NK1-RA + 5HT3-RA + dexamethasone ± olanzapine, can reduce the chances of anticipatory CINV in subsequent cycles.

Benzodiazepines for treating and preventing anticipatory CINV: Benzodiazepines are the recommended medication to treat and prevent anticipatory CINV [7,8,11]. They are especially useful to manage the anxiety associated with the treatment. The two main medications in this class are lorazepam and alprazolam; sedation is the most frequent side effect [24]. The experts in our study reached a "near consensus" regarding the use of benzodiazepines as an anxiolytic for managing anticipatory CINV. More than 50% of the expert oncologists in our study responded that "lorazepam" is their drug of choice for controlling anticipatory nausea and vomiting.

CINV Prophylaxis: Pharmacotherapy

The cornerstone pharmacologic classes employed in preventing and managing CINV encompass 5HT3-RA, NK1-RA, and corticosteroids. Additionally, adjunctive agents such as dopamine antagonists, benzodiazepines, cannabinoids, and the atypical antipsychotic drugs like olanzapine are utilized, albeit to a lesser extent [6,22]. Employing diverse mechanisms of action, these agents are often administered in combination regimens to achieve optimal antiemetic control, particularly in patients undergoing HEC or MEC protocols [22].

Triple or Quadruple Combination for CINV Prophylaxis

Per the international guidelines, the current pharmacological treatment for CINV following HEC chemotherapy includes either a triple or four-drug regimen containing 5HT3-RA, NK1-RA, dexamethasone, and/or olanzapine. Furthermore, for patients receiving MEC, guidelines suggest a 5HT3-RA with dexamethasone for CINV prophylaxis and the NK1-RA + 5HT3-RA + dexamethasone combination in certain patients with high‐risk characteristics for CINV or for whom prior 5HT3-RA + dexamethasone treatment has failed [6,20]. Experts reached a consensus recommendation that both triple-drug regimens and four-drug regimens should be used to treat acute CINV in patients receiving HEC. However, a median Likert scale score of 5.0 was obtained for the triple-drug regimen, compared to a score of 4.0 for the four-drug regimen in managing acute CINV. Therefore, in India, the triple-drug combination is preferred over the quadruple-drug combination for CINV prophylaxis.

Antiemetic Drugs

Neurokinin 1 receptor antagonists (NK1-RA): NK1-RA are a widely used class of antiemetic agents, which includes aprepitant, fosaprepitant, rolapitant, and netupitant as approved agents. NK1-RA are not typically employed as standalone antiemetic agents for acute CINV but instead are commonly utilized in combination with a 5HT3-RA and dexamethasone to optimize antiemetic efficacy [22]. NK1-RA has improved the complete response (CR) rate, i.e., no vomiting and no rescue medications needed, for the overall, delayed, and acute phase of emesis following HEC/MEC protocols [25-28]. Similar results are also observed with NK1-RA agents in the Indian population [29-32].

CINV prophylaxis of HEC: Studies have suggested that adding NK1-RA to the triple-drug regimen was more efficacious and safer than dual therapy (5HT3-RA + dexamethasone) for patients receiving HEC regimens [29,30]. Patients undergoing anticancer therapy with a heightened risk of emesis may require the inclusion of an NK1-RA to mitigate the risk further. Any NK1-RA can be incorporated into the four-drug regimen on day one, alongside a 5HT3-RA, dexamethasone, and olanzapine for patients receiving HEC regimens [7].

Netupitant (300 mg) is formulated as an oral fixed-dose combination product (NEPA) with palonosetron (0.5 mg) for managing acute and delayed CINV associated with HEC/MEC regimens. Numerous studies (clinical trials and real-world studies) have proven the effectiveness and safety of NEPA in preventing acute and delayed CINV, suggesting its role in improving adherence to guidelines [21,33-35]. In Indian settings also, a single dose of oral NEPA, which targets two different pathways, effectively managed the acute, delayed, and overall phases of CINV in patients on HEC and MEC regimens [36-38]. Experts gave a consensus recommendation that "netupitant is the preferred NK1 antagonist for CINV prophylaxis in triple-drug regimen."

There is a scarcity of trials comparing the effectiveness of different NK1-RA regimens. Thus, international guidelines and oncologists recommend all NK1-RAs as interchangeable and equal. In addition to simultaneously targeting two essential antiemetic pathways, Netupitant distinguishes itself from aprepitant due to its extended half-life. While aprepitant typically has a half-life of 9-13 hours, netupitant, a component of NEPA, boasts a longer half-life ranging from around 90 hours in blood, and netupitant also achieves >90% NK1 receptor occupancy in the striatum for around 96 hours. This prolonged elimination and half-life, shared by oral netupitant and palonosetron, may contribute to sustained protection against CINV, extending to 120 hours [39]. A pooled analysis of three phase 2/3 cisplatin-based HEC trials compared a single dose oral NEPA with a three-day aprepitant regimen (APR). Oral netupitant and palonosetron proved more effective than a three-day APR to manage delayed and overall CINV (0-120 hours) and provided better complete protection (defined as complete response plus absence of significant nausea) (delayed = 73.1% vs. 64.9%, p<0.05; overall = 69.4% vs. 61.8%, p<0.05). NEPA was also significantly more effective in preventing significant nausea in the delayed (79.8 vs. 73.7%; p = 0.049) as well as overall (78.3 vs. 71.4%; p = 0.031) phase of emesis, which is the most difficult-to-control symptom with cisplatin-based HEC. This may be attributed to the longer half-life of NEPA [40]. A good consensus was also reached by the Indian oncologists regarding the benefit of NEPA over the three-day APR.

5HT3 receptor antagonists: Different agents employed in the class of 5HT3-RA include first-generation agents such as ondansetron, dolasetron, and granisetron, having a half-life of around 3-9 hours, effective for controlling acute CINV. However, second-generation agents such as palonosetron have a half-life of 40 hours, making them effective in acute and delayed CINV. According to a meta-analysis of 16 randomized controlled trials, palonosetron emerged as a superior 5HT3-RA in efficacy and safety compared to other agents within its class [41]. Palonosetron has demonstrated high selectivity, with robust binding affinity and an extended plasma elimination half-life. Its effectiveness in preventing CINV has been demonstrated in both HEC and MEC settings, often in combination with other medications such as netupitant [21,35]. Furthermore, compared to ondansetron, palonosetron demonstrated superior clinical efficacy for the management of delayed (CR: 70.8% vs. 86.8%) and overall (CR: 65.1 vs. 82.1%) emesis and similar efficacy for acute (CR: 89.6% vs. 80.2%) emesis in an observational study conducted among South Indians [42]. The NCCN guidelines prefer palonosetron as the preferred 5HT3-RA in the triple-drug regimen [7]. Experts agreed on a consensus recommendation that "palonosetron is the preferred 5HT3 receptor antagonist for CINV prophylaxis in triple-drug regimen."

CINV prophylaxis of MEC: The NCCN guideline recommends the addition of NK1-RA to a 5HT3-RA/dexamethasone dual-drug regimen for patients undergoing a MEC regimen, especially those who possess additional risk factors or have experienced treatment failure with the two-drug regimen [7]. A pragmatic randomized, open-label study (PRAKYFRA trial) demonstrated the non-inferiority of NEPA to the three-day APR and reported a greater CR rate for NEPA (64.9%) compared to aprepitant (54.1%) in preventing CINV for patients receiving the MEC regimen [43]. Experts recommend the use of a triple-drug regimen (NK1-RA + 5HT3-RA + dexamethasone) to control delayed CINV in patients receiving moderately emetogenic chemotherapy (MEC), in order to improve treatment outcomes."

Dexamethasone Sparing Regimen

Dexamethasone or corticosteroids have long been a cornerstone in CINV management. Despite its generally accepted safety profile, even short-term dexamethasone administration is linked to diverse adverse events [9,44]. The guidelines have thus restricted the use of dexamethasone to a minimum in HEC/MEC regimens. Dexamethasone is recommended to be administered on days 2-4 in HEC settings [7]. Additionally, some evidence indicates that concurrent steroid use might diminish the effectiveness of immunotherapies. A study has reported that high-dose dexamethasone promotes breast cancer tumor growth and metastases [45]. Another study also reported tumor proliferation gemcitabine resistance and metastasis induced by dexamethasone in pancreatic cancer patients [46]. Thus, controlling the dose of dexamethasone in HEC/MEC settings is essential.

Single dose of 8 to 12 mg dexamethasone on day one with NEPA: A randomized non-inferiority study employing a simplified regimen of single dose of dexamethasone along with NEPA on day one depicted comparable CINV prevention throughout the five days of chemotherapy with cisplatin regimen along with the advantage of sparing patients' additional doses of dexamethasone as suggested by guidelines [44]. Furthermore, the OLNEPA study showed that excluding dexamethasone during CINV management with the HEC regimen is possible, and results are comparable to the guideline-recommended four-drug regimen (netupitant, palonosetron, olanzapine, and dexamethasone) [47]. Another open-label, phase IIa study depicted that administration of every-other-day NEPA without the use of dexamethasone was very effective in controlling CINV in patients at high risk of CINV receiving multiday high-dose chemotherapy for relapsed and refractory non-Hodgkin's lymphoma. This study provides a simplified antiemetic prophylactic approach in heavily pretreated and immunocompromised patients, which also spares the use of dexamethasone [48]. Experts agreed on a recommendation that NEPA with a single dose of dexamethasone effectively controls CINV for patients receiving MEC/HEC regimes, thus sparing the patients who are intolerant to corticosteroids.

Replacing dexamethasone with olanzapine in patients who do not tolerate steroids: Olanzapine is a dopamine receptor antagonist used widely as an antipsychotic. The NCCN/ASCO guidelines recommend the NK1-RA + 5HT3-RA + dexamethasone regimen ± olanzapine for HEC/MEC regimen [7,49]. Moreover, the recently updated guideline by MASCC/ESMO suggests that patients receiving multiple-day HEC regimens should receive a triple-drug regimen with olanzapine. Also, olanzapine may be beneficial for managing breakthrough CINV [50]. Many studies on Indian patients have revealed the beneficial effects of adding olanzapine to CINV prophylaxis, with higher CR rates in patients receiving olanzapine with the triple drug therapy [51-54].

Most of the guideline-based antiemetic regimens comprise dexamethasone as an essential component. However, all patients on anticancer regimens may not be able to tolerate dexamethasone, especially diabetic patients or patients on immunotherapy. Thus, a randomized, multicenter phase III trial was conducted evaluating low-dose olanzapine (5 mg) as an alternative to dexamethasone for patients receiving a cisplatin-based regimen. The results depicted a non-inferior CR rate for overall (83.6 vs. 84.9%, p=0.003) and delayed (85.5% vs. 85.6%, p = 0.024) phases of emesis. Furthermore, the side effects such as constipation, hiccups, and insomnia were also significantly fewer in the olanzapine group compared to the dexamethasone group [55,56]. The oncologists (experts) gave a "near consensus" recommendation on replacing olanzapine with dexamethasone for patients who could not tolerate it. And could not reach a full consensus on the same. This may highlight the difference in clinical practice in India.

CINV prophylaxis for multiday chemotherapeutic regimen: Managing multiday chemotherapy presents unique challenges due to potential differences in the mechanism and pattern of CINV compared to single-day regimens. Consequently, the effectiveness of antiemetic agents observed in single-day chemotherapy may not directly apply to multiday treatment. Limited data exist regarding the efficacy and safety of various agents in this specific context, leaving patients undergoing multiday regimens vulnerable to acute and delayed CINV [7,8].

Upto three days multiday chemotherapeutic regimen: The guidelines recommend administering an antiemetic regimen on each day of chemotherapy as well as two days after the regimen. In patients receiving HEC, a triple-drug regimen consisting of 5HT3-RA, NK1-RA, and dexamethasone is recommended for patients receiving multiday chemotherapy up to three days [7,8]. However, the studies with aprepitant + palonosetron/granisetron + dexamethasone have shown insufficient efficacy for the same [57]. A pilot study evaluating single-dose NEPA + dexamethasone showed significant benefit in sarcoma patients undergoing multiday chemotherapy regimens [58]. Corroborating this data, we also found that consensus among Indian oncologists for the beneficial use of single-dose NEPA in multiday chemotherapy regimens up to three days.

Five days or more multiday chemotherapeutic regimen: Netupitant (300 mg) is a potent agent that depicts >90% receptor occupancy in the striatum within 6 hours of administration and maintains this concentration for up to 96 hours, after which it starts to decline [59]. Thus, it was hypothesized that a repeat dose of netupitant may be required in patients receiving multiday (five-day) HEC regimens. We gathered the opinions of the Indian oncologists for the same and received a fair consensus. A recent randomized pragmatic study depicted the benefit of a single dose of NEPA over a prolonged period (144 hours) in patients on the MEC regimen [39]. However, the same beneficial effect in patients undergoing the HEC regimen is unknown and needs further studies.

## Conclusions

The current consensus study indicates that netupitant and palonosetron, along with dexamethasone, are the preferred antiemetic regimen for CINV prophylaxis in the Indian population. Most of the statements in the study achieved full consensus, while none fell into the category of no consensus. Near consensus was seen only for the use of lorazepam for anticipatory nausea and vomiting and the use of olanzapine instead of dexamethasone. However, there is a need for the development of robust evidence-based guidelines specific to the Indian population.

The implementation of CINV guidelines will enable oncologists to make informed and tailored decisions regarding CINV management across diverse healthcare settings. Moreover, adhering to the most effective approach in the individual patient for prevention and management of this side effect will facilitate the appropriate utilization of antiemetic agents and, consequently, enhance the overall quality of life for chemotherapy patients.
